# Is there anybody out there: what do senior surgeons expect of their youngsters?

**DOI:** 10.1515/iss-2018-0031

**Published:** 2019-02-01

**Authors:** Daniel Vallböhmer, Hans Fuchs, Ronny Dittmar, Carsten J. Krones

**Affiliations:** Klinikum Niederrhein, Duisburg, Germany; Department of General, Visceral and Tumor Surgery, University Hospital of Cologne, Cologne, Germany; Professional Association of German Surgeons, Berlin, Germany; Department of General, Visceral and Minimal Invasive Surgery, Marienhospital Aachen, Aachen, Germany

**Keywords:** career plan, senior surgeons, working hours, work-life balance, young surgeons

## Abstract

Surgery is indeed one of the most fascinating medical professions. However, it is also a stressful field of work with a high workload, and often leaves little time for personal and family needs. Within the last decade, a noticeable decline occurred in the willingness of medical students to enter a surgical residency. In fact, Generation Y is highly interested in a medical career with a respectful working atmosphere and balanced work and private life, as published in several recent papers. Therefore, surgery must evolve with the times to retain its attractiveness as a career choice for medical students and to compete for the best talents from Generation Y. However, little is known about what senior surgeons really expect from young surgical residents. On the basis of a recent survey by the Professional Association of German Surgeons, this paper tries to give some insights in this very relevant topic and a perspective on how to increase the attractiveness of our fascinating specialty. In fact, in this survey, senior surgeons defined a very clear requirement profile for surgical residency applicants. While the colleagues defined accurate applicant documents, a previous internship, self-motivation, and impressions from the job interview as the most important factors for a successful application for a surgical residency, a standard period of study or a dissertation was deemed of lower importance.

## Introduction

Surgery is indeed one of the most fascinating medical specialties. However, it is also a stressful profession with a high workload, and often leaves little time for personal and family needs.

Within the last years, a noticeable decline occurred in the willingness of medical students to enter a surgical residency. Already in 2003, Azizzadeh et al. assessed the factors influencing career choice among medical students interested in surgery [1]. The authors revealed in a survey with 111 medical students that the factors predicting surgery as a career choice were career opportunities and prestige, whereas lifestyle during residency, work hours, and quality of patient/physician relationships were all significantly negatively correlated with the choice of a surgical career [1]. Similar data were recently collected also in German institutions. For example, Kasch et al. performed a nationwide online survey in 9079 medical students from all German medical faculties, with questions related to future career choices and work satisfaction, as well as questions dealing with reasons for not working in patient care [2]. Their correlation analysis with “reasons for not working in patient care” revealed that work-life balance, career, professional needs, and working atmosphere had high priority. In addition, Kleinert et al. conducted an internet-based survey among 1098 medical students at two representative German university hospitals to gain more information about the underlying mechanisms that lead to opting for or against a surgical career [3]. While most students described surgery as an interesting and meaningful profession, >80% of them were not willing to choose a surgical specialty. The main reasons for this low motivation to choose a surgical profession were the demand of planning reliability, a sufficient work-life balance, flexibility in working hours and an existing childcare program, as well as a respectful conversional tone and a steady appreciation of the individual work. The authors suggested that adjustments to working hour models, clinical curriculum, and a respectful interaction are factors that might increase the motivation of medical students to choose a surgical profession [3]. Finally, just recently, Roeth and Mille raised in their paper the question of what young surgeons prioritize in their professional life, and tried to describe the modern requirements for senior surgeons [4]. The colleagues highlighted the importance of a defined curriculum, assistance of sub-steps during surgery, residency dialogues held on a regular basis, logbooks, the possibility of training and simulation in the clinic, as well as permission to participate in further education and training in a structured and transparent residency. Moreover, they underlined that this structured residency has to go along with the compatibility of family and work, better planning of the routine in the clinic, a positive feedback culture, as well as a balanced relationship of work and life.

## Survey of the Professional Association of German Surgeons 2018: “Are we now taking everybody?”

As shown above, surgery must evolve with the times to retain its attractiveness as a career choice for medical students and to compete for the best talents from Generation Y. However, little is known about what senior surgeons really expect from young surgeons these days, and what is the current situation of applicants for residency in surgical departments. With these findings, we could ideally bring the expectations of young and senior surgeons together in order to overcome the current low attractiveness of the surgical profession.

Therefore, the Professional Association of German Surgeons recently performed a questionnaire survey among senior surgeons about the current situation of applicants for residency in their surgical departments, and explored their expectations from the young colleagues [5]. The survey included 715 (12.3% of the respondents) senior surgeons (head of the department, 38.6%; senior consultant, 61.4%) mainly working in general/abdominal (41.7%) or trauma (30.3%) surgery.

**Figure 1:**
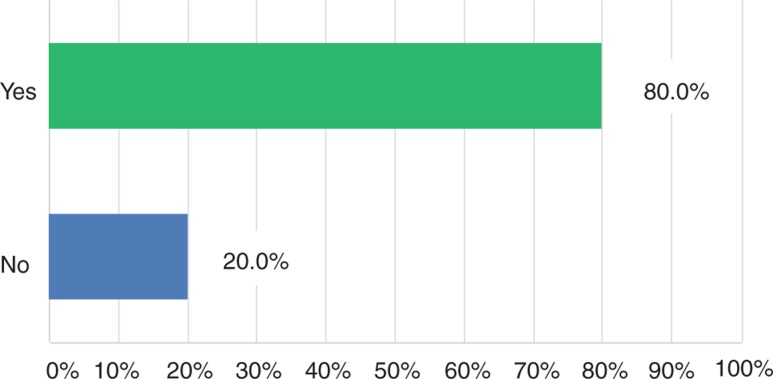
Do you believe that there is a decline of applicants for surgical residency?

**Figure 2:**
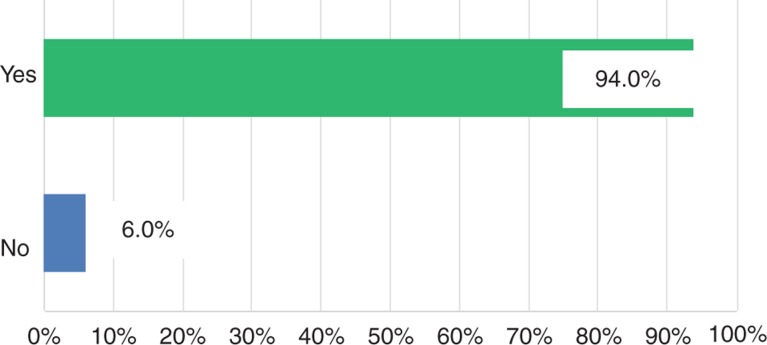
Do you believe that there is a quality deficit in the remaining candidates?

**Figure 3:**
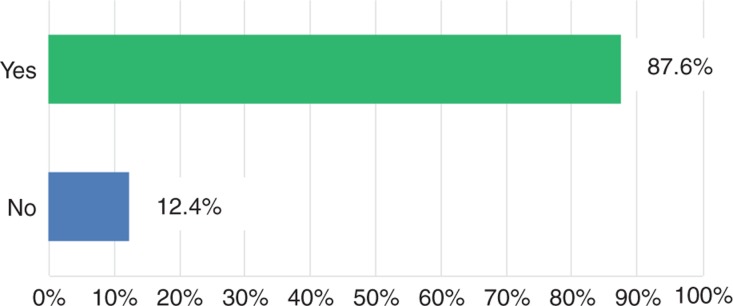
Due to the lack in number and quality of surgical residents, do you predict negative consequences in the quality of supply in future surgery?

The vast majority of senior surgeons (80%) reported a huge decline of applicants for surgical residency (Figure 1). Moreover, the participants of the survey complained about a quality deficit in the remaining candidates (94%, Figure 2). Finally, due to the lack in number and quality of surgical residents, most of the senior surgeons (87.6%) predicted negative consequences in the quality of supply in future surgery (Figure 3). These results are indeed very alarming. However, what are the reasons for such a poor applicant situation in the surgical profession? In the current survey, there were different explanation attempts [5]. Some senior surgeons attributed the low acceptance of surgical residency to the current admission rules for medical school in Germany. Others criticized the ongoing rigid and tense hierarchies as well as brusqueness in surgical departments, leading to the rejection of the Generation Y of the surgical profession. Finally, many senior surgeons mentioned the poor integration of surgical residents with immigrant background in the medical system as being responsible for the low number and quality of applicants in surgical departments.

Finally, the senior surgeons of this survey defined a very clear requirement profile for surgical residency applicants [5]. While the colleagues defined accurate applicant documents, a previous internship, self-motivation, and impressions from the job interview as the most important factors for a successful application for surgical residency, a standard period of study or a dissertation was deemed of lower importance.

## Perspectives for the future

### So, is there anybody out there?

In fact, within the last years, a noticeable decline occurred in the willingness of medical students to enter a surgical residency. However, we still believe that Generation Y is highly interested in a medical career. However, a respectful atmosphere, a balanced workload, and a satisfying private life have to be secured (Table 1). Under these circumstances, the new generation of surgical candidates is still willing to perform hard in the current demanding working environment. Therefore, surgery must evolve with the times to retain its attractiveness as a career choice for medical students and to compete for the best talents from Generation Y.

**Table 1:** Expectations of young and senior surgeons about requirements in a surgical career.

Young surgeons	Senior surgeons
Respectful working atmosphere	Self-motivation
Sufficient work-life balance	Achievement of a high standard of surgical performance
Flexibility in working hours	Previous internship
Defined curriculum/residency dialogues	Accurate applicant documents

What is the situation in other countries? Is this just a German problem? In fact, the findings in other countries are very similar. For example, Ellison et al. just recently predicted a huge shortage of general surgeons to meet the future demand in the United States [6]. Similar findings were made by Reid-Lombardo et al. in a survey by the Society for Surgery of the Alimentary Tract about the future shortage of surgeons [7]. Moreover, the authors described generational differences in responses to contributors and solutions for the impending shortage. While senior surgeons placed a high value on improving reimbursement, tort reform, and surgeon burnout, surgical residents held a strong interest in a national loan forgiveness program and improving lifestyle barriers.

However, senior surgeons currently describe a lack in number and quality of surgical residents and apprehend negative consequences in the quality of supply in German surgery. Nevertheless, senior surgeons define a clear requirement profile for surgical residency applicants (Table 1). Moreover, they are not willing to ease these conditions, especially to maintain the high standard of surgical performance in Germany.

On first view, these attitudes seem to be contradictory. However, to work hard and properly and to live enjoyably at the same time do not exclude each other. To the authors’ opinion, structured training programs, reliable career plans, and modern working hours represent the promising keys to solve this problem. High-class surgery is not only a question of time. However, the requirements of a surgical profession defined by the senior surgeon based on a tremendous wealth of experience have to be respected as well. Accordingly, an ongoing exchange of expectations and requirements defined by both generations needs to be started.

## Supplementary Material

The article (iss-2018-0031) offers reviewer assessments as supplementary material.
