# Supplementary material for: Is there anybody out there: what do senior surgeons expect of their youngsters?
Source: Innov Surg Sci. 2019 Feb 1;4(1):3–6. doi: 10.1515/iss-2018-0031 (PMC6754063; doi:10.1515/iss-2018-0031)
Supplement: Supplementary file 1 [file iss-4-20180031-s001.pdf]

Reviewer Assessment

Daniel Vallböhmer*, Hans Fuchs, Ronny Dittmar and Carsten J. Krones

Is there anybody out there: what do senior surgeons expect of their youngsters?

https://doi.org/10.1515/iss-2018-0031
Received September 22, 2018; accepted December 28, 2018

***Corresponding author: Daniel Vallböhmer,** Klinikum Niederrhein, Duisburg, Germany, E-mail: daniel.vallboehmer@evkln.de

Reviewers' Comments to Original Submission

Reviewer 1: anonymous

Sep 25, 2018

**Reviewer Recommendation Term:**	Revise with Major Modifications
**Overall Reviewer Manuscript Rating:**	60
**Custom Review Questions**	**Response**
Is the subject area appropriate for you?	5 - High/Yes
Does the title clearly reflect the paper's content?	3
Does the abstract clearly reflect the paper's content?	2
Do the keywords clearly reflect the paper's content?	2
Does the introduction present the problem clearly?	3
Are the results/conclusions justified?	3
How comprehensive and up-to-date is the subject matter presented?	3
How adequate is the data presentation?	1 - Low/No
Are units and terminology used correctly?	N/A
Is the number of cases adequate?	N/A
Are the experimental methods/clinical studies adequate?	N/A
Is the length appropriate in relation to the content?	3
Does the reader get new insights from the article?	2
Please rate the practical significance.	3
Please rate the accuracy of methods.	N/A
Please rate the statistical evaluation and quality control.	N/A
Please rate the appropriateness of the figures and tables.	N/A
Please rate the appropriateness of the references.	4
Please evaluate the writing style and use of language.	4
Please judge the overall scientific quality of the manuscript.	2
Are you willing to review the revision of this manuscript?	Yes

Open Access. © 2019 Vallböhmer D., et al., published by De Gruyter. This work is licensed under the Creative Commons Attribution 4.0 Public License.

Comments to Authors:

This paper requires some modifications. The topic is of utmost significance, but even if I consider the manuscript as an opinion paper, some data should be presented, although they may have been published elsewhere.
In detail:
- the authors should state in the abstract what senior surgeons expect from young surgeons
- the keywords should include: career plan, work life balance, working hours; delet: generation y
- the data of the survey should be presented: how many senior surgeons answered? percentages of answers should be given, e.g. drop of applicants; what deficits were complained of? what do senior surgeons predict for the future? the explanation attempts should be quantified as well, the reference alone is not sufficient enough
the chapter "discussion" should be named "perspectives for the future", because it is not really a discussion; also, for example, a table with recommendations for senior surgeons to get along with the actual requirements of young surgeons would be helpful
Additionally, one or two international references would be helpful: how do other countries handle the problem? what do other national societies offer for senior surgeons? what are the fears of other countries with respect to future surgical patients' care? The authors should present - if possible - some more infromation on this relevant issue.

Reviewer 2: Tim Pohlemann

Oct 17, 2018

**Reviewer Recommendation Term:**	Accept
**Overall Reviewer Manuscript Rating:**	N/A
**Custom Review Questions**	**Response**
Is the subject area appropriate for you?	5 - High/Yes
Does the title clearly reflect the paper's content?	5 - High/Yes
Does the abstract clearly reflect the paper's content?	4
Do the keywords clearly reflect the paper's content?	4
Does the introduction present the problem clearly?	4
Are the results/conclusions justified?	3
How comprehensive and up-to-date is the subject matter presented?	5 - High/Yes
How adequate is the data presentation?	3
Are units and terminology used correctly?	4
Is the number of cases adequate?	2
Are the experimental methods/clinical studies adequate?	N/A
Is the length appropriate in relation to the content?	5 - High/Yes
Does the reader get new insights from the article?	3
Please rate the practical significance.	N/A
Please rate the accuracy of methods.	N/A
Please rate the statistical evaluation and quality control.	N/A
Please rate the appropriateness of the figures and tables.	N/A
Please rate the appropriateness of the references.	3
Please evaluate the writing style and use of language.	5 - High/Yes
Please judge the overall scientific quality of the manuscript.	3
Are you willing to review the revision of this manuscript?	Yes

Comments to Authors:
Interesting new data supporting the "general discomfort" about the quantity and quality of present appliants for surgical careers. The article is not a classic scientific paper, but due to the possible "political Impact" the results should be published as a basis for ongoing and deeper analyses.

Authors' Response to Reviewer Comments

Nov 18, 2018

Reviewer#1

Reviewer #1: This paper requires some modifications. The topic is of utmost significance, but even if I consider the manuscript as an opinion paper, some data should be presented, although they may have been published elsewhere.

In detail:

- the authors should state in the abstract what senior surgeons expect from young surgeons

We additionally stated in the abstract what senior surgeons expect from young surgeons.

- the keywords should include: career plan, work life balance, working hours; delet: generation y

The keywords were changed as suggested.

- the data of the survey should be presented: how many senior surgeons answered? percentages of answers should be given, e.g. drop of applicants; what deficits were complained of? what do senior surgeons predict for the future? the explanation attempts should be quantified as well, the reference alone is not sufficient enough

Additional data of the survey was added to the paper.

- the chapter “discussion” should be named “perspectives for the future”, because it is not really a discussion; also, for example, a table with recommendations for senior surgeons to get along with the actual requirements of young surgeons would be helpful

The changes were made as suggested.

- Additionally, one or two international references would be helpful: how do other countries handle the problem? what do other national societies offer for senior surgeons? what are the fears of other countries with respect to future surgical patients‘ care? The authors should present - if possible - some more information on this relevant issue.

Additional references were added and discussed in paper.

Reviewer #2:

Interesting new data supporting the “general discomfort” about the quantity and quality of present applicants for surgical careers. The article is not a classic scientific paper, but due to the possible “political Impact” the results should be published as a basis for ongoing and deeper analyses.

No additional corrections were made based on the reviewer comments.

Reviewers' Comments to Revision

Reviewer 1: anonymous

Nov 05, 2018

**Reviewer Recommendation Term:**	Accept
**Overall Reviewer Manuscript Rating:**	N/A
**Custom Review Questions**	**Response**
Is the subject area appropriate for you?	5 - High/Yes
Does the title clearly reflect the paper's content?	5 - High/Yes
Does the abstract clearly reflect the paper's content?	5 - High/Yes
Do the keywords clearly reflect the paper's content?	5 - High/Yes
Does the introduction present the problem clearly?	5 - High/Yes
Are the results/conclusions justified?	5 - High/Yes
How comprehensive and up-to-date is the subject matter presented?	5 - High/Yes
How adequate is the data presentation?	4

Are units and terminology used correctly?	N/A
Is the number of cases adequate?	4
Are the experimental methods/clinical studies adequate?	N/A
Is the length appropriate in relation to the content?	5 - High/Yes
Does the reader get new insights from the article?	4
Please rate the practical significance.	4
Please rate the accuracy of methods.	3
Please rate the statistical evaluation and quality control.	N/A
Please rate the appropriateness of the figures and tables.	4
Please rate the appropriateness of the references.	5 - High/Yes
Please evaluate the writing style and use of language.	4
Please judge the overall scientific quality of the manuscript.	4
Are you willing to review the revision of this manuscript?	Yes

Comments to Authors:
All questions were answered and critical aspects were taken care of. The paper ist now - at least to my opinion - ready for publication.

Reviewer 2: Tim Pohlemann

Dec 27, 2018

**Reviewer Recommendation Term:**	Accept
**Overall Reviewer Manuscript Rating:**	N/A

**Custom Review Questions**	**Response**
Is the subject area appropriate for you?	5 - High/Yes
Does the title clearly reflect the paper's content?	5 - High/Yes
Does the abstract clearly reflect the paper's content?	5 - High/Yes
Do the keywords clearly reflect the paper's content?	5 - High/Yes
Does the introduction present the problem clearly?	5 - High/Yes
Are the results/conclusions justified?	5 - High/Yes
How comprehensive and up-to-date is the subject matter presented?	5 - High/Yes
How adequate is the data presentation?	N/A
Are units and terminology used correctly?	N/A
Is the number of cases adequate?	N/A
Are the experimental methods/clinical studies adequate?	N/A
Is the length appropriate in relation to the content?	5 - High/Yes
Does the reader get new insights from the article?	5 - High/Yes
Please rate the practical significance.	5 - High/Yes
Please rate the accuracy of methods.	N/A
Please rate the statistical evaluation and quality control.	N/A
Please rate the appropriateness of the figures and tables.	N/A
Please rate the appropriateness of the references.	4
Please evaluate the writing style and use of language.	5 - High/Yes
Please judge the overall scientific quality of the manuscript.	3
Are you willing to review the revision of this manuscript?	Yes

Comments to Authors:
Unchanged opinion, interesting paper of General Impact on a question of Junior surgeons.
